# Role of Perfusion Index and Pulse Variability Index in the Assessment of Neonatal Hemodynamics: A Systematic Review

**DOI:** 10.7759/cureus.48058

**Published:** 2023-10-31

**Authors:** Vinod Kumar Mandala, Suresh Babu Mendu, Suresh Kumar Yadav Bollaboina, Rakesh Kotha

**Affiliations:** 1 Department of Pediatrics, Kakatiya Medical College, Warangal, IND; 2 Department of Pediatrics, Government Medical College, Siddipet, IND; 3 Department of Pediatrics, Niloufer Hospital, Hyderabad, IND; 4 Department of Neonatology, Osmania Medical College, Hyderabad, IND

**Keywords:** non-invasive hemodynamic monitoring, noninvasive haemodynamic monitoring, neonatal shock, neonatal hemodynamics, pulse variability index, perfusion index

## Abstract

Hemodynamic monitoring of neonates is crucial because neonates are easily and acutely susceptible to hemodynamic disturbances. As such, non-invasive monitoring of hemodynamics is preferable. It has been postulated that non-invasive pulse oximetry determines the perfusion index and pulse variability index and provides accurate measurements to predict hemodynamic changes in preterm or term infants. Equally, numerous studies have investigated the efficacy of perfusion and pulse variability indices in monitoring neonatal hemodynamics.

The aim of this study was to systematically review studies that have delved into the role of perfusion and pulse variability indices in the assessment of neonatal hemodynamics. The study collected data from 2010-2023 using the patient, intervention, comparison, outcome (PICO) search strategy using the databases PubMed, Scopus, and Excerpta Medica database (Embase). A total of 616 articles were evaluated based on their appropriateness and relevance; we included seven studies. As per the Preferred Reporting Items for Systematic Reviews and Meta-Analyses (PRISMA) guidelines, we conducted a systematic review. Our study concluded that these indices were effective in measuring hemodynamics.

## Introduction and background

Newborns, especially preterm infants, are at a high risk for morbidity and mortality, especially in the first weeks of life, because of health complications associated with neonatal hemodynamics [1]. Most of the neonatal health complications, such as severe neonatal sepsis, which accounts for 11%-27% of neonatal intensive care unit admissions, are associated with high mortality rates [1]. Neonatal intensive care units are essential to the well-being of preterm infants as they allow healthcare professionals to monitor neonatal hemodynamics. Monitoring neonatal hemodynamics is critical as it helps in assessing and managing the cardiovascular systems of newborns. However, early assessment and diagnosis of neonatal health complications such as neonatal sepsis represent one of the main challenges neonatologists face. Perfusion index and pulse variability index are essential early indices that can help healthcare professionals in neonatal intensive care units monitor the circulation of blood in newborn infants, or rather the status of neonatal hemodynamics, to inform medical interventions required to reduce morbidity and mortality risks [2].

Perfusion index is a measurement that reflects the amplitude of the blood flowing through the peripheral tissues, especially capillaries. The perfusion index is provided by the pulse oximeter. Cresi et al. (2010) argued that the pulse oximeter has become a reliable tool among neonatologists as it helps them monitor changes in the flow of peripheral blood [1]. The perfusion index, a measurement derived from the signal of the pulse oximeter, reflects real-time changes in the flow of peripheral blood. In particular, the pulse oximeter provides neonatologists with an oximeter waveform that shows changes in the flow of peripheral blood. Neonatologists use the level of the waveform to establish the inadequate perfusion associated with critically ill newborn infants. Low perfusion index values indicate high illness in newborn infants. A PI lower than 1.24 has been used as a threshold to diagnose a baby with a severe neonatal illness [2].

Gupta and Donn (2020) argued that the perfusion index is a non-invasive method that neonatologists can easily use to monitor early changes in the postnatal hemodynamic conditions of newborn infants [3]. Reference perfusion index values are currently available [4]. In most cases, perfusion index values are expressed as percentages (0.02%-20%), making it easy for neonatologists to track neonatal hemodynamics. Another advantage of the perfusion index is that it is unambiguous, offers more clarity, and is independent compared to other measurements for assessing neonatal hemodynamics [4]. Gupta and Donn (2020) argued that neonatologists can objectively predict the status of neonatal hemodynamics or illness severity among newborn infants when they combine the perfusion index and pulse variability index [3].

The pulse variability index is a measurement or signal that reflects the amplitude or variations in the arterial pulse. Like the perfusion index, the pulse variability index is measured non-invasively using the pulse oximeter. A pulse oximeter is a device that neonatologists or other healthcare professionals use to monitor the heart rate and the saturation of oxygen based on the flow of fluids [5]. Neonatologists derive signals from the pulse reflecting changes in perfusion index from the pulse oximeter to establish blood pressure, heart rate, and arterial pressure. Pulse pressure variation (PPV) and stroke volume variation (SVV) have been the most common methods used to predict fluid load, heart rate, and arterial pressure [5]. Monitoring fluid status and heart rate using these methods presents significant challenges to healthcare professionals since they require invasive monitoring. In particular, neonatologists find establishing invasive monitoring of newborn children challenging. With the pulse variability index, neonatologists find it easy and convenient to monitor neonatal hemodynamics non-invasively. The pulse variability index calculates hemodynamic changes using minimum and maximum perfusion index values over a predetermined period. Yiğit et al. (2018) argued that the pulse variability index offers neonatologists an opportunity to detect hemodynamic changes occurring in spontaneously breathing newborns. In addition, the pulse variability index gives healthcare professionals the latitude to conduct cesarean sections as they are guaranteed to track neonatal hemodynamic changes or respiratory morbidity risks associated with them. In their investigation of the effectiveness of perfusion index and pulse variation index after cesarean section, Yiğit et al. (2018) found that perfusion index and pulse variation index were able to show significant hemodynamic changes in newborn infants in their first hour of life [2]. We planned this review because of the importance of the topic and the lack of reviews in the presently available sources.

## Review

Method

Search Strategy

Literature on the role of perfusion index and pulse variability index in the assessment of neonatal hemodynamics was systematically searched, retrieved, and reviewed to collect evidence and relevant information and statistics that address the role of perfusion index and pulse variability index in assessing neonatal hemodynamics. Cooper et al. (2018) indicated that a literature search is an integral and important exercise when conducting a systematic review of the literature [6]. A search strategy entails looking for articles that address the research question or topic. In view of the critical role played by the literature search, the literature search focused on identifying valid and evidence-binding articles that offered transparent findings regarding the role of the perfusion index and pulse variability index in the assessment of neonatal hemodynamics. Articles and literature addressing the role of perfusion index and pulse index in the assessment of neonatal hemodynamics were retrieved from databases that host journals, articles, and report papers in the field of medical sciences, such as Cumulative Index to Nursing and Allied Health Literature (CINAHL), Science Direct, PubMed, Scopus, the National Library of Medicine, and Excerpta Medica database (Embase).

The search process followed the population, intervention, comparison, and outcome (PICO) approach as a guide, leading to the most appropriate articles or literature addressing the research topic. The rationale for using the PICO approach entails its high precision in locating articles, as it specifies searches. In using the PICO approach, the search involved combining population, intervention, comparison, and outcome elements. The population encompassed newborn infants or preterm children. The intervention encompassed the perfusion index and the pulse variability index. The comparison was marked by measurement methods other than the perfusion index and pulse variability index, while the outcome was marked by non-invasive, easy-to-use, and conventional ways of monitoring neonatal hemodynamics. The implementation of the PICO approach followed a combination of population, intervention, comparison, and outcome elements, a truncation, or interchanging of elements to meet contextual research needs (Table 1).

**Table 1:** PICO framework used in the review PI: perfusion index; PVI: pulse variability index

PICO Framework	
Population	Neonates
Intervention	PI and PVI
Comparison	Other standard and invasive methods
Outcome	Monitoring of hemodynamics by non-invasive methods (PI and PVI)
Study	Observational studies

Secondly, the search process considered synonyms, related terms, and acronyms to improve the quality of the search. For example, the acronym "PVI" was used in place of the "pulse variability index" to improve the quality of the search. The search exercise also involved using Boolean operators, especially 'AND', to combine keywords of the research topic. Keywords such as perfusion index and pulse variability index were used in various combinations with neonatal hemodynamics. The search was refined to retrieve recent articles that address the research question. Table 2 illustrates the structure of search components and keywords used to identify articles from online databases that address the research topic.

**Table 2:** Search strategy MeSh: Medical Subject Headings

S.No. & Search	Search Strategy
#1. Search	Assessment of neonatal dynamics (MeSh terms)
#2. Keywords search	Perfusion index and pulse variability index in the assessment of neonatal hemodynamics (Title/Abstract)
#3. Keyword search	Pulse variability index in the assessment of neonatal hemodynamics (Truncation)
#4. Keyword search	Role of perfusion index in the assessment of neonatal hemodynamics (Truncation)
#5. Keyword search	Assessment of neonatal perfusion (Truncation)
#6. Keyword search	Perfusion index and pulse variability index in newborn infants (Alternative combinations, alternative terms)
#7. Keyword search	Role of perfusion index and pulse variability index in assessment of neonatal hemodynamics (Title/Abstract)

Identification of the Studies

All article citations were retrieved using the EndNote tool (Clarivate, London, UK). The retrieved articles were screened manually to remove duplicates. The screening process also involved picking appropriate articles that addressed the research topic. Appropriate articles were screened by following the content of their titles, abstracts, key findings, conclusions, and full text. The search process yielded 616 potential articles related to the research topic. External reviewers reviewed three articles to confirm whether they met the threshold to be included in the systematic review of the literature. Articles identified by peer-reviewed articles initially identified were also accepted to be used in the systematic review of the literature.

Data Extraction and Retrieval

The inclusion/exclusion criteria for the present study followed the criteria illustrated in Table 3.

**Table 3:** Inclusion and exclusion criteria for the systematic review

Inclusion Criteria	Exclusion Criteria
Findings from at least one random controlled trial study that was peer-reviewed	Articles with abstracts only
Findings from cohort studies, pilot studies, or systematic reviews	Editorial commentary articles
Findings from a non-randomized trial study that was peer-reviewed	Conference papers
Studies focusing directly on perfusion index and pulse variability index in the assessment of neonatal hemodynamics	Articles focusing on perfusion index or pulse variability index in the adult population.

Results

**Figure 1:**
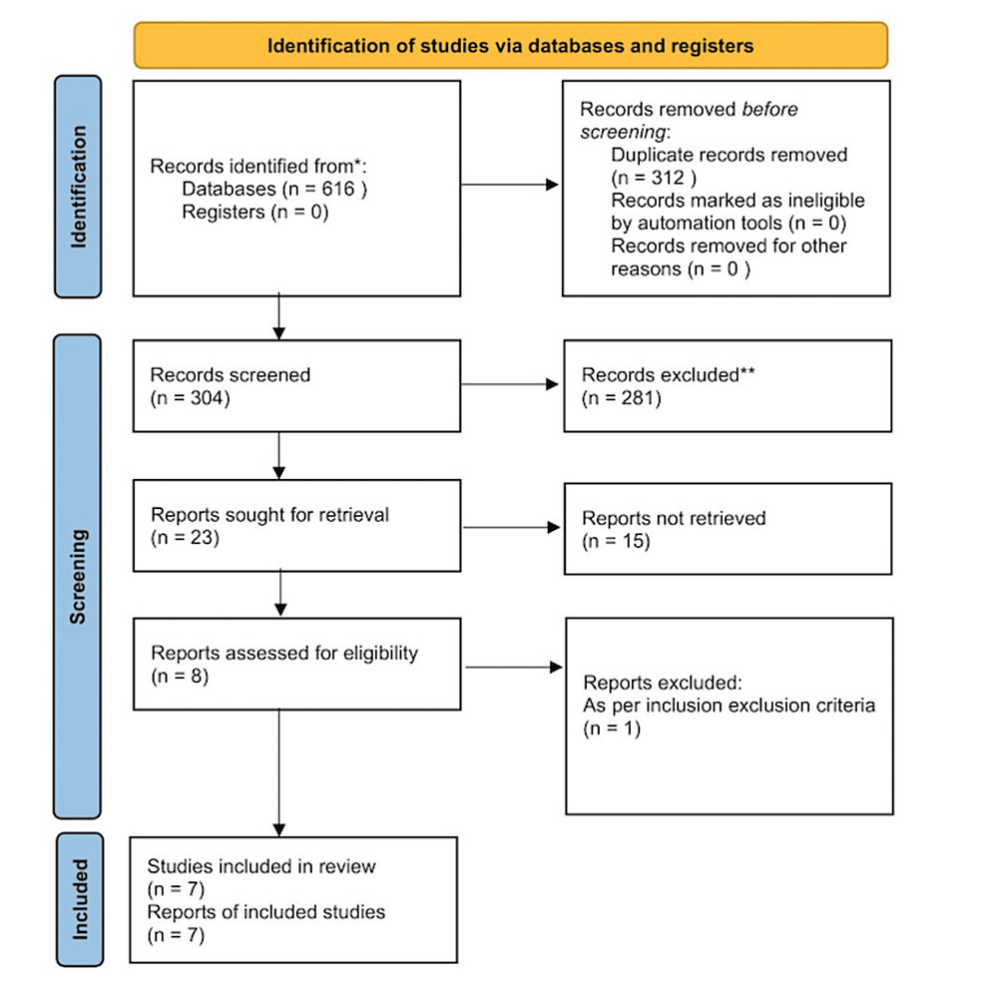
PRISMA flow diagram to showcase the study selection process PRISMA: Preferred Reporting Items for Systematic Reviews and Meta-Analyses

The initial research produced 616 articles presumed to address the research topic. After review, a total of 312 duplicates were disregarded, leaving 304 articles subject to further review. Further review based on relevance rendered 281 articles irrelevant or inappropriate. The remaining 23 articles were filtered based on how directly they addressed the research topic, leaving 8 articles. Based on a careful examination informed by inclusion/exclusion criteria, one article was excluded, leaving seven articles that were approved to have met the selection criteria (Figure 1).

Quality Assessment

The quality assessment of the studies was done using the Newcastle-Ottawa scale. The Newcastle-Ottawa scale is an effective tool for assessing the quality of studies. The tool helps analyze the strengths and biases held by each study, making it easy to identify appropriate and effective studies that can be used in a systematic review. The Newcastle-Ottawa scale grades the quality of studies based on group selection, comparability of the groups, and outcome. The selection encompasses four sections, namely, representatives of the study, selection of the study, ascertainment of exposure, and demonstration regarding the outcome of interest. Each of these aspects was awarded a maximum of one star. Comparability contained three questions seeking to establish study controls for socioeconomic status (SES), for any additional factor, or for an inadequate degree of control. Each question was assigned a maximum of one star (Table 4).

**Table 4:** Quality ratings of the selected articles based on the Newcastle-Ottawa scale

SN	Study	Selection	Comparability	Outcome
1	Yiğit et al., (2017)(2)	***	**	***
2	Hua et al. (2023) [7]	***	**	***
3	Cresi et al. (2010) [1]	***	**	***
4	Bagci et al. (2013) [8]	***	**	***
5	Plana et al. (2018) [9]	***	**	***
6	Alderliesten et al. (2015) [10]	***	**	***
7	Piasek et al. (2014) [11]	***	**	***

The outcome section entailed assessing the quality of the outcome by establishing whether it was backed by secure records, independent studies, or observations. It also entailed establishing record linkages, cases of self-report, especially where no references were made to original medical records, or establishing outcomes where no descriptions were given. Each aspect of this assessment was awarded a maximum of one star.

Description of Individual Studies

This systematic review established that both the perfusion index and the pulse variability index play an integral role in assessing neonatal hemodynamics. Yiğit et al. (2017) investigated the efficacy of both the perfusion index and the pulse variability index in assessing hemodynamic changes in newborn babies born with spontaneous vaginal delivery and cesarean sections. The study found that both the perfusion index and pulse variability index were higher in newborns born via cesarean sections. The high perfusion index and pulse variability index values in newborns born via cesarean sections were indicative that cesarean sections are associated with significant neonatal hemodynamic changes [2]. Hua et al. (2023) investigated the effectiveness of the perfusion index in healthy and stable newborns between six and 72 hours of life subjected to different altitudes. The study argued that differences in the perfusion index values generated from newborns do not mean that the perfusion index is an ineffective measurement for neonatal hemodynamics [7]. Differences in perfusion index values were attributable to the varying altitudes that the newborns were exposed to. At sea level, the perfusion index has the ability to generate uniform results because of the equal atmospheric pressure experienced by newborns. The study concluded that the perfusion index is an effective and efficient measurement for assessing neonatal hemodynamics. It is recommended that neonatologists and health professionals, in general, consider the reference ranges of perfusion index at different ranges when assessing neonatal hemodynamics.

Bagci et al. (2013) conducted a non-randomized pilot study that investigated the role of the pulse variability index in detecting hemodynamics regarding volume-responsive hypotension in newborn infants. The study found that the pulse variability index had a diagnostic value regarding hemodynamic status in newborn infants [8]. In particular, Bagci et al. (2013) established that the pulse variability index had 86% sensitivity for volume-responsive hypotension. In addition, only one of the one hundred and three-volume expansions evaluated failed to meet the criteria for volume-responsive hypotension. Volume expansion refers to the first therapy given to a child or any patient when arterial hypotension occurs [8].

Plana et al. (2018), in their systematic review of controlled trial studies, showed that pulse oximetry had a sensitivity of 76.3% and a specificity of 99.9% in screening for congenital heart defects in newborns [9]. The study concluded by arguing that pulse oximetry was more effective in screening congenital heart defects in newborn infants than the main screening methods, especially prenatal ultrasonography and postnatal clinical examination.

Equally, Cresi et al. (2010) investigated the effectiveness of the perfusion index in assessing hemodynamic changes in clinically stable preterm newborns in a children’s hospital in Turin. The study showed that the perfusion index is an effective measurement for monitoring hemodynamic changes in preterm newborns. Secondly, the study pointed out how the non-invasive nature of the perfusion index made it convenient to monitor the hemodynamics of preterm newborns [1]. In addition, Cresi et al. (2010) concluded that neonatologists should use the perfusion index in measuring neonatal hemodynamics given its ability to detect hemodynamic status in newborns in the first week of life. A cohort study by Alderliesten et al. (2015) investigated the value of the perfusion index in monitoring neonatal hemodynamics in preterm infants in the first three days of life. The study established that the perfusion index has the potential to monitor neonatal dynamics as well as provide guidance for medical interventions [10]. Using 24 hours to 72 hours as the best fit, Alderliesten et al. (2015) found that the perfusion index was lowest in the hours below the 18-hour mark of life. A steady rise in the value of the perfusion index was witnessed in the hours of life beyond the 18 mark, indicating its proactiveness in measuring neonatal hemodynamics [10]. In addition, Piasek et al. (2014) found that the perfusion index is a valuable measurement for monitoring neonatal hemodynamics. Through a systematic review, the study showed that the perfusion index meets the threshold required in the assessment of neonatal hemodynamics [11].

Discussion

The purpose of this systematic review was to determine the role of the perfusion index and pulse variability index in the assessment of neonatal hemodynamics. Studies regarding the role of perfusion index and pulse variability index in the assessment of neonatal hemodynamics have been conducted. Yet discourse concerning their efficacy in assessing neonatal hemodynamics still exists.

This study is one of the current systematic reviews that consolidates evidence from various studies of varying nature, including but not limited to randomized trials, non-randomized trials, and case-control studies, to present a holistic and comprehensive argument that elucidates the role of the perfusion index and pulse variability index in the assessment of neonatal hemodynamics (Table 5).

**Table 5:** Summary of the selected articles

	Authors	Gestational age	Newborns age	Condition/morbidity
1	Yiğit et al. (2017) [2]	38.5±1.09 weeks	One hour of life after a cesarean section	Hemodynamic changes
2	Hua et al. (2023) [7]	More than 28 weeks	Six to 72 hours of life	Clinical and hemodynamic stability, asymptomatic well newborns
3	Cresi et al. (2010) [1]	28 and 36 weeks	First week of life	Hemodynamically stable
4	Bagci et al. (2013) [8]	All gestational ages	During surgery	Volume-responsive hypotension
5	Plana et al. (2018) [9]	All gestational ages	Up to 24 hours of life	Congenital heart defects
6	Alderliesten et al. (2015) [10]	Less than 32 weeks	First three days of life	Hemodynamic changes
7	Piasek et al. (2014) [11]	All gestational ages	Sick neonates	Clinical and hemodynamic stability

Newborns' skin perfusion is higher than their oxygen demand under normal conditions, which is why in critical situations, cardiac output is redistributed to provide oxygen to critical organs like the heart, brain, and adrenal glands. Monitoring the perfusion index can be valuable in assessing a newborn's condition as in neonates, the skin is the largest organ with a major blood supply [12].

These indices are more valuable in neonatal screening for congenital heart diseases and early detection of babies who were exposed to maternal chorioamniotis [13]. As per sources, we found only one review on the perfusion index, which reviewed 25 articles and noted that the perfusion index is more valuable in the clinical assessment of neonates [14]. A thorough meta-analysis of patients of all ages discovered that the pulse variability index had a reasonable ability to predict fluid responsiveness [15].

The systematic review found that both the perfusion index and pulse variability index have an assessment and diagnostic value in the monitoring of neonatal hemodynamics. With the concept of pulse oximetry, all studies with certainty on perfusion and pulse variability indices have the potential to assess the clinical conditions of newborn infants regarding hemodynamic conditions.

The efficacy of perfusion and pulse variability indices has been clinically proven. Both indices are indirect and non-invasive and provide continuous assessment of neonatal hemodynamics, which is crucial in providing useful health information for reducing morbidity and mortality among newborn infants [16]. Perfusion and pulse variability indices are derivatives of pulse oximetry. Pulse oximetry makes it easy for health professionals to monitor hemodynamic changes in newborn infants and make informed decisions regarding their health status [17-19]. The selected articles agree that a low perfusion or pulse variability index is an indicator of severe illness in newborn infants. Pulse oximeter values need to be standardized as per gestational age.

## Conclusions

The results of this systematic review indicate that perfusion and pulse variability indices are effective measurements that can enable neonatologists to monitor hemodynamic changes occurring in newborn infants. Both indices are easy to apply. In addition, they are non-invasive methods of assessing early hemodynamic changes in newborn infants who cannot handle invasive methods. Users of perfusion and pulse variability indices should consider factors that can alter standard readings of perfusion and pulse variability indices, such as altitude, to accurately track hemodynamic changes in newborn infants.
